# Differentiation Potential of CD14^+^ Monocytes into Myofibroblasts in Patients with Systemic Sclerosis

**DOI:** 10.1371/journal.pone.0033508

**Published:** 2012-03-14

**Authors:** Nadine Binai, Steven O'Reilly, Bridget Griffiths, Jacob M. van Laar, Thomas Hügle

**Affiliations:** 1 Musculoskeletal Research Group, Institute of Cellular Medicine, Newcastle University, Newcastle upon Tyne, United Kingdom; 2 The Freeman Hospital, Newcastle upon Tyne, United Kingdom; 3 Department of Rheumatology, Felix-Platter-Spital, University of Basel, Basel, Switzerland; Universität Würzburg, Germany

## Abstract

**Background:**

Circulating monocytes are a highly plastic and functionally heterogeneic cell type with an activated phenotype in patients with systemic sclerosis (SSc). CD14^+^ monocytes have the potential to differentiate into extra-cellular matrix (ECM) producing cells, possibly participating in fibrogenesis.

**Aim:**

To study the effect of GM-CSF, IL-4 and endothelin -1 (ET-1) alone or in combination on monocyte differentiation into myofibroblasts.

**Methods:**

CD14^+^ cells were isolated from peripheral blood from 14 SSc patients and healthy controls by positive selection and incubated with different combinations of GM-CSF, IL-4 and ET-1 for 14 days. Type-1 collagen and α-SMA were detected by Western blot, qPCR and confocal microscopy. HLA-DR, CD11c and CD14 expression was analysed by flow cytometry. A collagen gel contraction assay was performed for functional myofibroblast assessment.

**Results:**

GM-CSF both induced collagen and α-SMA expression after 14 days. ET-1 further increased GM-CSF-induced collagen expression in a dose dependent manner up to 30-fold. IL-4/GM-CSF combination leads to a more DC-like phenotype of monocytes associated with reduced collagen and α-SMA expression compared to GM-CSF alone. Collagen and α-SMA expression was higher in monocytes from SSc patients and monocytes were more prone to obtain a spindle form. In contrast to controls, ET-1 and IL-4 alone were sufficient to induce α-SMA expression in monocytes from SSc patients. Despite the induction of α-SMA expression, monocyte-derived myofibroblasts only had a moderate capability of contraction in functional analyses.

**Conclusion:**

SSc monocytes display increased maturation towards myofibroblasts demonstrated by their phenotype and α-SMA expression when compared to monocytes from healthy controls, however only with minor functional contraction properties.

## Introduction

Systemic sclerosis (SSc) is an autoimmune multisystem connective disease where low grade inflammation and vasculopathy lead to increased extracellular matrix (ECM) deposition in affected tissues, notably the skin, lungs and gastrointestinal tract [1], [2]. Inflammation in SSc is characterized by the infiltration of monocytes, notably in the early phase of the disease [3]. CD14^+^ monocytes are bone marrow derived, highly plastic and functionally heterogeneous cells. Apart from their well known capacity to differentiate into dendritic cells (DC) and macrophages, they also have the potential to differentiate into collagen-producing fibrocytes [4] or other cells sharing characteristics of cells with mesenchymal or ectodermal origin [5]. In SSc patients, circulating CD14^+^ monocyte numbers are increased, with an activated status demonstrated by their expression of CD68, CD204 and Siglec-1 [6]–[8]. Collagen-expressing CD14^+^CD34^+^ fibrocytes have been found in higher numbers in lungs of patients with SSc-associated interstitial lung disease [9] and fibrocyte recruitment to the skin has been demonstrated in the bleomycin mouse model [10].

Myofibroblasts are key effector cells in fibrosis by their capacity to synthesize collagen and contractile α-smooth muscle actin (SMA). Myofibroblasts differentiate either directly from fibroblasts e.g. under the influence of TGF-beta or Endothelin-1 (ET-1) [11], [12], from epithelial cells via epithelial mesenchymal transition (EMT) [13] or from fibrocytes [14]. In atherosclerosis models it has been demonstrated that haematopoietic cells can differentiate into α-SMA producing cells, actively promoting intima fibrosis [15].

Granulocyte-macrophage colony-stimulating factor (GM-CSF) is the main differentiation factor for monocytes [16]. GM-CSF is produced by a variety of cell types including T- and B-lymphocytes, macrophages and endothelial cells [17]. The combination of GM-CSF in combination with IL-4 is commonly used to differentiate monocytes into DC. GM-CSF is required for *in vitro* monocyte survival, proliferation and as trigger of macrophage differentiation, whereas the combination with IL-4 drives differentiation of monocytes into DC [18]. *In vitro* culture of monocytes in the presence of GM-CSF for a prolonged time stimulates the formation of CD14^low^ cells with a DC morphology and high viability, expressing HLA-DR, CD86 and CD11a but low CD1a [19]. These cells are refractory to maturation induced by inflammatory stimuli, thus showing a more regulatory character than classical, IL-4 driven inflammatory DC. Upon subcutaneous injection, GM-CSF leads initially to the infiltration of macrophages; after chronic administration, GM-CSF induces fibrotic responses with accumulation of α-SMA -expressing myofibroblasts [20]. GM-CSF levels rise during inflammation [21] and are higher in bronchoalveolar lavage fluid from patients with idiopathic pulmonary fibrosis compared to healthy controls [22]. Similar to the described overexpression of GM-CSF-receptors on SSc fibroblasts [23], GM-CSF–receptors are also upregulated on mononuclear cells infiltrates in scar formation [24].

ET-1 plays an important pathogenic role in SSc. ET-1, which is highly expressed in vessels, acts as vasoconstrictor but is also known for its function as differentiation factor [25]. The effect of ET-1 has mainly been studied on fibroblasts where ET-1 both stimulates α-SMA production and collagen matrix contraction. In SSc, ET-1 is overexpressed by endothelial cells and fibroblasts, as compared to healthy controls [26].

We hypothesised that activated monocytes from SSc patients have a dysregulated differentiation potential upon stimulation with GM-CSF, IL-4 and also ET-1 into ECM producing, myofibroblast-like cells, thereby participating in fibrosis.

## Materials and Methods

### Patients

Peripheral blood was collected from 14 patients with limited (n = 9) or diffuse (n = 5 ) SSc (1 male and 13 females; age 45–83 years, mean ± SD 62.2±12.3 years). The mean disease duration was 8.6 years (range 2–20 years). Six patients were on treatment with immunosuppressive medication. Patient characteristics are summarized in Table 1. Control blood samples were obtained anonymously from the blood center Newcastle in accordance with ethical regulations in the UK. The protocol was approved by Newcastle University Joint Research Office and the National Research Ethics Service and written informed consent has been obtained from the patients.

**Table 1:** Patient characteristics.

Age/Gender	Disease subtype	Disease duration (years)	Auto-Antibody	Immuno-supessive treament
77/f	Limited	12	ANA	-
68/f	Limited	1	ACA	AZA
60/f	Diffuse	2	ANA	-
57/f	Limited	7	ACA	-
60/f	Limited	2	ANA	-
64/f	Diffuse	11	nd	nd
62/f	Limited	11	nd	-
45/f	Limited	11	ACA	-
63/m	Diffuse	5	-	Cyc, MMF
72/f	Limited	17	ACA	-
38/f	Diffuse	2	-	Cyc, MMF
83/f	Limited	11	-	Penicillamine
50/f	Limited	7	-	MTX
60/f	Diffuse	20	Scl-70	Cyc

Abbreviations: f:female, m:male, ANA: anti-nuclear antibodies; ACA: anti-centromere antibodies; nd: no data, AZA, azathioprine, Cyc: cyclophosphamide, MMF: mycophenolate mofetil, MTX: methotrexate.

### Monocyte isolation and cultivation

PBMCs were isolated from peripheral blood from SSc patients or anonymised healthy controls (n = 8) using Lymphoprep™ (Axis-Shield, Olso, Norway). Monocytes were isolated from the PBMC fraction using CD14 microbeads (Miltenyi, Bergisch-Gladbach, Germany). Monocytes were seeded into tissue culture plates in a density of 1×10^6^ cells per ml and treated for 14 days with 50 ng/ml GM-CSF, 50 ng/ml IL-4 (both Immunotools, Friesoythe, Germany) or 0–500 ng/ml ET-1 (Calbiochem, Merck, Darmstadt, Germany). The culture medium was replenished every 3 days. Light microscopy was used to observe morphological changes. For quantification, spindle shaped cells per 5×5 cm field were counted and normalized to the cell number.

### Western blotting

Total cell extracts were prepared in 20 mM Tris (pH 7.5), 1 mM EDTA, 100 mM NaCl, 1% Trition X-100, 0,5% Desoxycholate, 0,1% SDS and Protease inhibtor cocktail (Roche, Welwyn Garden City, UK). Protein extracts were subsequently separated by SDS-PAGE on 4–20% gradient gels (Bio-Rad, Hercules, CA) and blotted on PVDF membranes. To detect proteins, Western blot analysis was performed using anti-type 1 collagen, anti-alpha SMA (both Millipore, Temecula, USA), anti-vimentin (DAKO) and anti-GAPDH antibodies (HyTest, Turku, Finnland). Western Blots were quantified by using ImageJ software.

### Confocal microscopy

For immunfluorescence staining 1.5×10^5^ monocytes were seeded in 8-well Culture slides (BD Falcon, Oxford, UK) and cultured for 14 days. After fixation with 4% Formaldehyde and permeabilisation with 0.02% Triton X-100 cells were blocked in 10% goat serum. Specimens were stained with anti-α-SMA (Millipore, Temecula, USA) and 4′,6-Diamidine-2′-phenylindole dihydrochloride (DAPI) (Roche Applied Science, Burgess Hill, UK) and mounted in Mowiol 4–88 (Sigma Aldrich, UK). For imaging of the slides a Leica SP2 AOBS UV confocal microscope with a 63× oil immersion objective was used.

### Procollagen expression

Quantitative assessment of mRNA expression levels was achieved by 2-step real-time PCR (qRT-PCR). Total RNA was extracted using the RNeasy Mini Kit (Qiagen, Crawley, UK). Reverse transcription of mRNA into cDNA from 0.5–1 µg of the extracted total RNA was conducted using the QuantiTect RT Kit (Qiagen). Subsequently, the cDNA was subjected to qRT-PCR in triplicate using the ABi HT 7900 system (Applied Biosystems, USA) and the nonspecific DNA binding dye SYBR green I for the detection of PCR products (SYBR Green real-time PCR Kit from Sigma Aldrich, UK). Procollagen was amplified with gene-specific oligonucleotide primer pairs (forward 5′-CAA GAG GAA GGC CAA GTC GAG G -3′, reverse 5′-CGT TGT CGC AGA CGC AGA T -3′). To quantify the relative expression levels of these genes, the expression of 18S mRNA was also determined in the same cDNA samples using a gene-specific primer pair (forward 5′ CGA ATG GCT CAT TAA ATC AGT TAT GG-3′ reverse 5′-TAT TAG CTC TAG AAT TAC CAC AGT TAT CC-3′).

### Collagen-gel contraction assay

Collagen gels were prepared as described elsewhere [27]. Briefly, 0.5×10^6^ monocytes were seeded on the collagen gels and stimulated as describes above with GM-CSF, IL-4 or ET-1. Pictures where take with the Syngene G:Box imaging system and the gel area was calculated using Image J software.

### Statistical analysis

All data were evaluated using Student t-test. For non-parametric analysis, Mann Withney U and Kruskal Wallis tests were applied. P-values<0.05 were considered statistically significant. Results are shown as mean and standard error of the means.

## Results

### CD14^+^ monocytes differentiate into type-1 collagen and α-SMA expressing cells in the presence of GM-CSF

**Figure 1:**
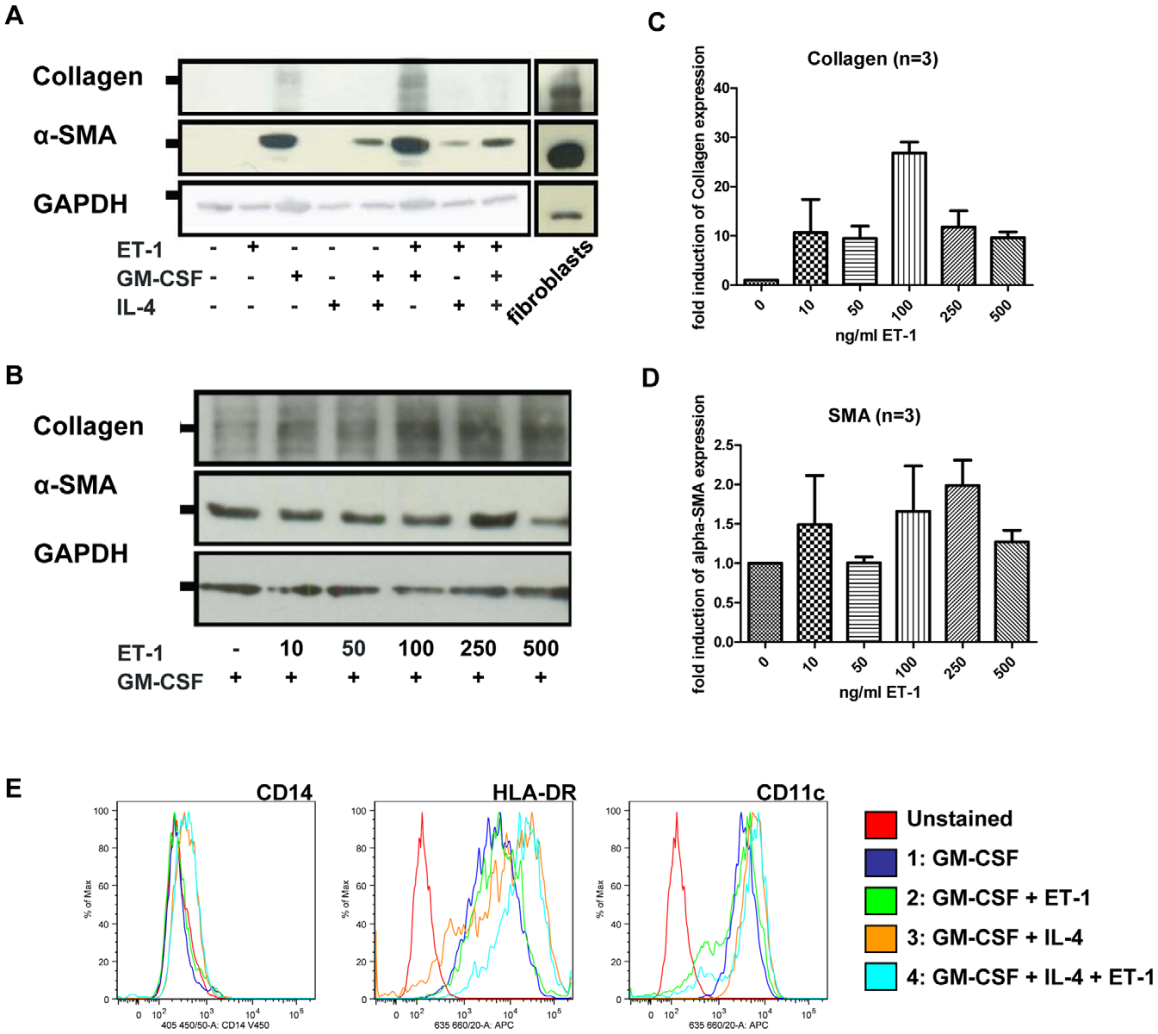
Healthy monocytes can differentiate into α-SMA and collagen expressing cells upon GM-CSF stimulation. A) CD14^+^ monocytes from healthy donors were cultured for 14 days with GM-CSF, IL-4 or ET-1. Western blot analysis showed expression of type-1 collagen and α-SMA under distinct conditions. Fibroblasts were used as a positive control, GAPDH was used as loading control. B) Monocytes were treated with GM-CSF and 0–500 ng/ml of ET-1 over 14 days. Western Blot analysis for type-1 collagen and α-SMA was conducted. GAPDH was used as loading control. C) and D) Quantification of the western blots from 3 independent experiments are shown. E) Expression of CD14, HLA-DR and CD11c was assessed by flow cytometry.

To study the differentiation potential of monocytes into myofibroblasts, the expression of α-SMA as a myofibroblast marker and type-1 collagen was determined by western blot analysis. Monocytes cultured for 14 days solely in medium and 10% FCS did not express collagen or α-SMA (Figure 1A). The presence of GM-CSF induced expression both of α-SMA and type-1 collagen. We detected collagen expression after 7 days and α-SMA after 14 days (data not shown). The addition of ET-1 to GM-CSF further increased collagen and to a lesser extent α-SMA expression, whereas IL-4 in addition to GM-CSF reduced α-SMA and prevented collagen expression. The combination of IL-4 and ET-1 without GM-CSF weakly induced α-SMA, but not collagen expression.

### ET-1 upregulates GM-CSF-induced type-1 collagen expression in a dose-dependent manner

We incubated CD14^+^ cells from healthy individuals for 14 days in the presence of 50 ng/ml GM-CSF and increasing doses of ET-1 from 0–500 ng/ml (Figure 1B). In relation to GAPDH, collagen expression increased in the presence of ET-1. The highest collagen levels corresponding a nearly 30-fold increased expression were observed at an ET-1 concentration of 100 ng/ml (Figure 1C). Conversely, ET-1 lead only to a 2-fold α-SMA expression (Figure 1D).

### Phenotype of cultured monocytes in the presence of GM-CSF, IL-4 and or IL-4

We analysed the phenotype of the monocytes after 14 days culture in the presence of different combinations of GM-CSF, ET-1 (100 ng/ml) and IL-4 by flow cytometry (Figure 1E). IL-4 with or without ET-1 added to GM-CSF lead to a higher expression of HLA-DR, and to a lesser extent also CD11c, as compared to GM-CSF alone or GM-CSF and ET-1. CD14 expression was lost during the time of culture albeit CD14 expression was partly preserved in the presence of IL-4, altogether suggesting a more DC-like phenotype in the presence of IL-4.

### Type-1 collagen and α-SMA expression is higher in monocytes from SSc patients

**Figure 2:**
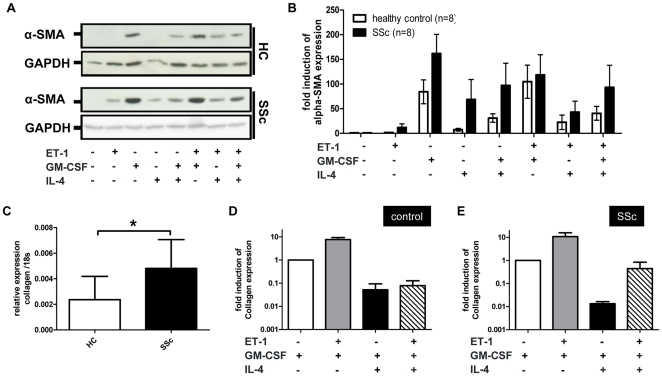
Patient monocytes need less stringent stimulation to upregulate α-SMA. A) Monocytes from healthy controls or SSc patients were cultured for 14 days with GM-CSF, IL-4 or ET-1. α-SMA levels were detected by Western blot analysis. GAPDH was used as loading control. B) Quantification of Western blots from 8 patients and 8 healthy controls showed stronger expression of α-SMA in SSc monocytes compared to healthy controls despite no significant difference could be detected. C) qPCR analysis of the procollagen expression in GM-CSF treated monocytes from healthy controls (HC) (n = 4) or SSc patients (n = 8). Data were normalized to expression of 18S. (p = 0.481). D) and E) Monocytes from HC (n = 4) and SSc patients (n = 3) were treated with various combinations of GM-CSF, IL-4 and ET-1. Procollagen expression was measured by qPCR. Data were normalized to expression of 18S. The concentration of ET-1 in all experiments was 100 ng/ml. * Statistically significant (P-value<0.05).

5×10^5^ CD14^+^ monocytes were isolated from patients with SSc and healthy controls and incubated for 14 days according previous experiments in medium supplemented with 10% FCS and different combinations of ET-1, GM-CSF and IL-4. GM-CSF-induced α-SMA expression appeared higher in samples from SSc patients (Figure 2A). In contrast to healthy samples, α-SMA was induced in SSc monocytes which were stimulated by ET-1 or IL-4 alone. ET-1/GM-CSF, IL-4/GM-CSF and ET-1/GM-CSF/IL-4 combination induced a higher α-SMA expression in SSc patients compared to healthy controls (Figure 2B), although no statistical significance could be detected between the subgroups.

Baseline expression of procollagen as measured by qPCR was only detected in the presence of GM-CSF. GM-CSF-induced collagen expression was significantly higher in SSc monocytes (p<0.05) (Figure 2C). The addition of ET-1 to GM-CSF resulted in a 10-fold increase collagen expression in both in monocytes from healthy controls (Figure 2D) and SSc patients (Figure 2E). Similar to our findings with α-SMA, the addition of IL-4 to GM-CSF also reduced collagen expression. We did not observe a difference in α-SMA or collagen expression between patients with limited or diffuse SSc subtypes (data not shown). As a third myofibroblast structural protein we determined vimentin expression by Western blotting in cultured monocytes. Unlike type-1 collagen and α-SMA, vimentin expression was observed in untreated monocytes after 14 (Figure S1). Similar to collagen and α-SMA, GM-CSF was the strongest factor for induction of vimentin expression, both in monocytes from SSc patients and healthy controls.

### Monocytes from SSc patients obtain a more spindled shape

**Figure 3:**
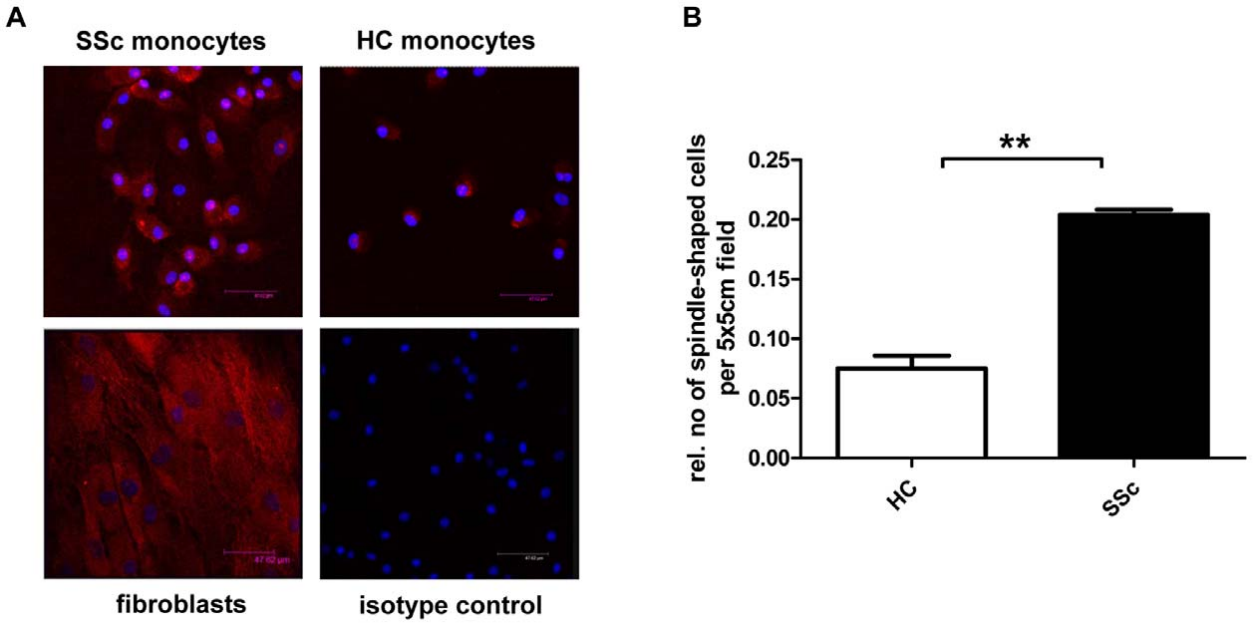
Morphological analysis of monocytes from SSc patients and healthy controls. A) Immunofluorescence staining of α-SMA (red) in GM-CSF treated monocytes from healthy control or a SSc patient. Fibroblasts were used as positive control. Nuclei were stained with DAPI (blue). For quantification, spindle shaped cells per 5×5 cm field were counted and normalized to cell numbers (B). ** Statistically highly significant (P-value<0.01).

For a morphological analysis, we stained healthy or SSc monocytes incubated with GM-CSF for 14 days with α-SMA antiserum for confocal microscopy. We found that monocytes from SSc patients expressed more α-SMA and obtained a more spindled shape than monocytes from healthy controls (Figure 3A). After normalization to cell numbers, the relative number of spindle shaped cells per 5×5 cm field was significantly higher in monocytes from SSc patients (p<0.001) (Figure 3B).

### Functional collagen-gel contraction anlaysis

**Figure 4:**
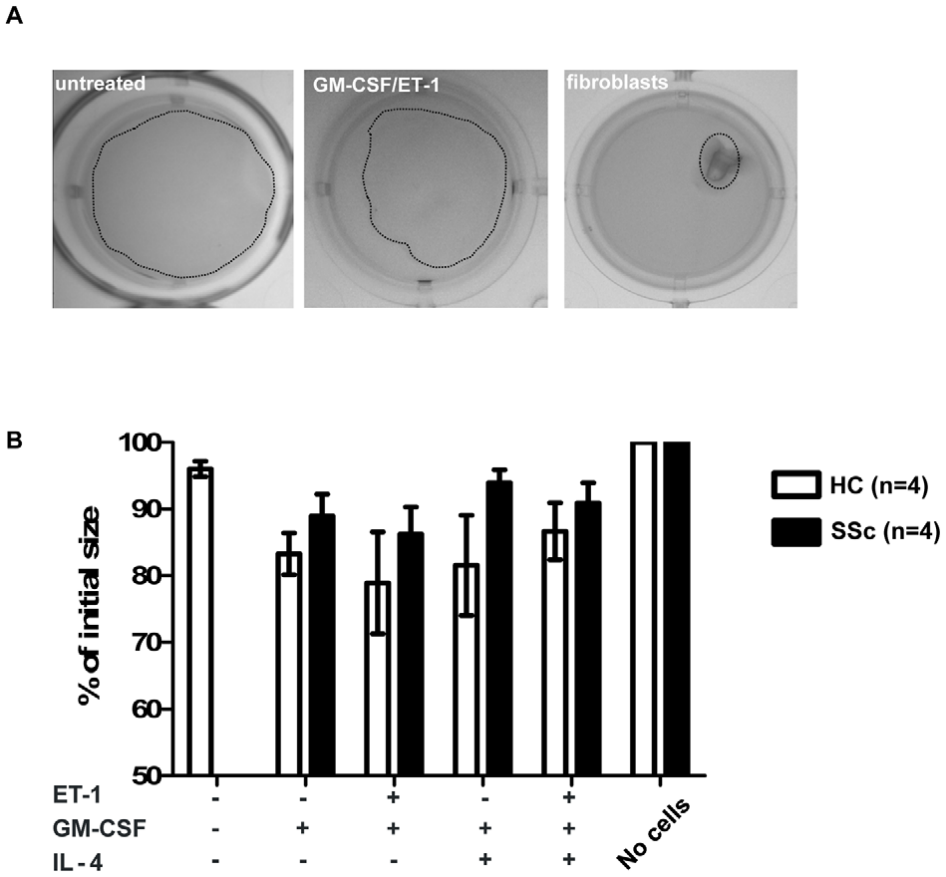
GM-CSF treated monocytes are partially contractile. 5×10^6^ CD14^+^ monocytes from SSc patients or healthy controls were seeded on collagen gels and cultured for 14 days under different conditions as indicated. On day 14, the reduction of the gel area was calculated as percent of the initial seize. B) The graph shows the summary of data from 4 healthy controls and 4 SSc patients. A significant difference between the two groups was not detectable.

For functional assessment of contraction, we seeded 5×10^5^ CD14^+^ monocytes on collagen gels under before mentioned conditions with GM-CSF, IL-4 or ET-1 (Figure 4A). Fibroblasts were used as positive controls and yielded a 97,6% contraction of the gel after 14 days. Monocyte-induced myofibroblasts contracted the collagen gel at a maximum of 30% whereas no contraction was observed without monocytes and a maximum of 5% contraction with untreated monocytes. The strongest contraction was observed in cells treated with ET-1 and GM-CSF (Figure 4B). No significant difference of contraction was detectable between healthy and SSc monocytes.

## Discussion

This proof-of-concept study demonstrates the differentiation potential of monocytes in the direction of myofibroblasts in SSc patients and healthy controls under the influence of GM-CSF, ET-1 or IL-4. Monocytes from SSc patients were more prone to differentiate into myofibroblasts, as demonstrated by significantly higher levels of collagen expression, spindle-shaped myofibroblast-like cell morphology and a trend towards higher α-SMA production, compared to healthy control monocytes. Matrix production of cultured SSc monocytes and the morphological difference provides further evidence that monocytes may play an important role in SSc fibrogenesis. We postulate that myofibroblasts, which are often encountered perivascularly in SSc tissue, are chemokine-attracted, extravasated cells potentially originating from the large pool of circulating monocytes. The previously described alternatively activated status of SSc monocytes and their impaired subset distribution in the peripheral blood might account for our observations. To this end, further studies will be necessary to clarify the differentiation potential of e.g. CD14^+^16^+^ monocyte subset into myofibroblasts.

To our knowledge, we provide the first evidence that the prolonged culture of healthy monocytes with GM-CSF exhibits collagen and α-SMA production after 14 days. Based on its recognized pathogenic effect in SSc, we added ET-1 as another cell differentiation factor to panel. By the analysis of GM-CSF, ET-1 and IL-4 alone and in combination we aimed to investigate a more physiological setting rather than observing the effect of single cytokines or differentiation factors. GM-CSF clearly turned out as the strongest inducer of α-SMA, collagen and also vimentin as a third cytoskeletal protein. GM-CSF-induced collagen expression was further stimulated by ET-1 in a dose dependent manner, but inhibited by IL-4. Similarly, in healthy individuals, GM-CSF-induced α-SMA expression was moderately stimulated by ET-1, but reduced in the presence of IL-4. In monocytes derived from SSc patients, and the presence of GM-CSF, both ET-1 and IL-4 slightly decreased α-SMA production. However, unlike in healthy monocytes, IL-4 and ET-1 alone or in combination induced α-SMA expression in SSc monocytes. The latter finding is in line with findings in fibroblasts, where IL-4 also has a stimulating effect on α-SMA expression [28] suggesting that monocytes-derived myofibroblasts from SSc patients share characteristics with fibroblasts. The inhibitory effect of IL-4 and ET-1 on α-SMA expression in combination with GM-CSF possibly is due competitive inhibition within subcellular signalling pathways, e.g. NF-kB, similar to observations in keratinocytes [29], or alternatively, a shift of their phenotype towards DC.

Phenotype analysis by flow cytometry revealed that monocytes cultured with IL-4 express higher of levels HLA-DR, and slightly higher levels of CD11c. CD14^+^ monocytes loose CD14 during culture, albeit to a lesser extent in the presence of IL-4. This confirms a more DC-like phenotype in an IL-4 condition, and thus might explain the lower α-SMA and collagen expression in this circumstance. As CD34^+^ expression has not been tested, we can not clarify to what extent monocyte-derived myofibroblasts under these conditions correspond to fibrocytes. Furthermore, we did not address in this study the question whether our observations are due to selective outgrowth of a monocyte subtype such as fibrocytes or CD16^+^ monocytes, or whether all monocytes are equally affected by an altered differentiation potential. It is likely that the increased amount of CD14^+^CD16^++^ monocytes observed in the elderly react differently on GM-CSF, IL-4 or ET-1 stimulation. The age difference between patients and healthy controls therefore could influence our observations. As a further limitation, monocytes from patients suffering from non-fibrosing disorders have not been analysed. A difference of apoptosis between healthy and SSc monocytes could underlie the reduced α-SMA expression in healthy controls; we therefore tested the difference of apoptosis in SSc patients versus healthy controls after 14 days of culture by flow cytometry and did not find a significant difference (data not shown). In our experiments, GM-CSF, IL-4 or ET-1 stimulation led to a different expression pattern between type-1 collagen and α-SMA. Further studies are needed to determine whether this reflects outgrowth of different cellular subsets. To confirm the functional relevance of monocyte differentiation we performed contractility assays which showed that monocyte-derived myofibroblasts result in a maximum of 30% contraction of collagen gelx, compared to 5% by the untreated monocytes. In comparison with a 95% contraction by fibroblasts, the contractile property of monocyte-derived myofibroblast is modest. We also could not detect a clear difference between monocyte derived myofibroblasts from SSc patients and healthy individuals, respectively. Possible explanations for this partial effect could be the lower size of the myofibroblasts compared to fibroblasts with an impaired fiber architecture and the necessity of a further contractile stimulus after the differentiation process. However, this finding also shows that the differentiation process from monocytes into myofibroblast under the tested conditions is not complete and that it might require additional stimulation or further differentiation *in vivo*.

Nevertheless, our findings do show a potential of monocytes to differentiate into spindle formed myofibroblasts with a higher expression of matrix proteins which suggest that albeit reduced functional properties, these cells are involved in SSc associated fibrosis, vasculopathy or skin contractions and that notably GM-CSF is a key player in this differentiation process. In future studies, the differentiation potential of monocytes in SSc has to be characterized by functional analyses and compared with the histological analysis and serum levels of GM-CSF, ET-1 and IL-4 in the same individuals.

## Supporting Information

Figure S1 **GM-CSF upregulates Vimentin in monocytes from SSc patients and healthy controls.** Monocytes from healthy controls or SSc patients were cultured for 14 days with GM-CSF, IL-4 or ET-1. Vimentin levels were detected by Western blot analysis. GAPDH was used as loading control.
(TIF)
