# Supplementary figures and images for: Differentiation Potential of CD14+ Monocytes into Myofibroblasts in Patients with Systemic Sclerosis
Source: PLoS One. 2012 Mar 14;7(3):e33508. doi: 10.1371/journal.pone.0033508 (PMC3303833; doi:10.1371/journal.pone.0033508)

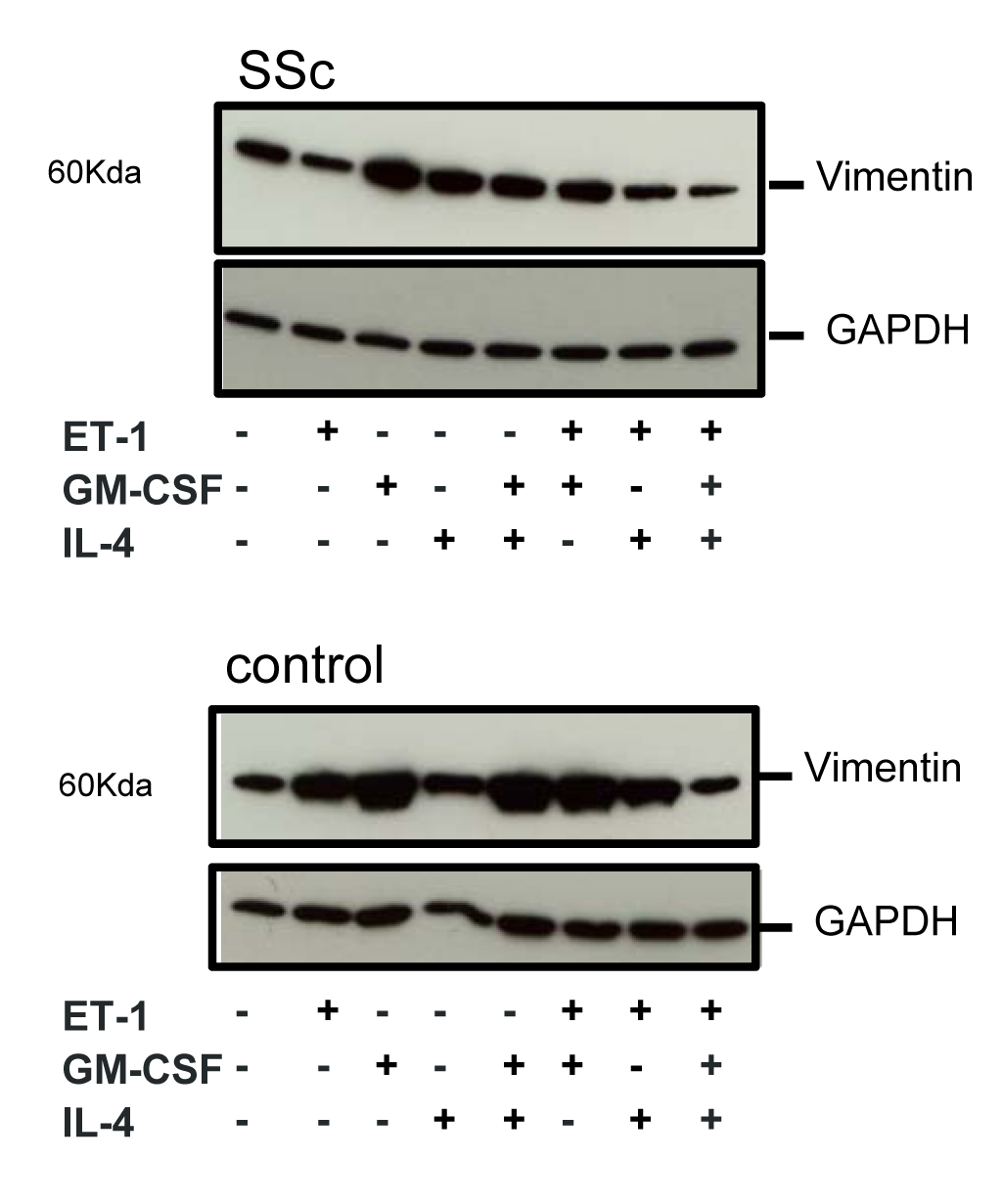

Supplement: Figure S1 — GM-CSF upregulates Vimentin in monocytes from SSc patients and healthy controls. Monocytes from healthy controls or SSc patients were cultured for 14 days with GM-CSF, IL-4 or ET-1. Vimentin levels were detected by Western blot analysis. GAPDH was used as loading control. (TIF) [file pone.0033508.s001.tif]
